# The Lullaby Project: A Musical Intervention for Pregnant Women

**DOI:** 10.1089/whr.2020.0084

**Published:** 2020-12-07

**Authors:** Jennifer Hinesley, Sarah Cunningham, Rashel Charles, Kirsten Olsen, Saba Masho, Susan Kornstein

**Affiliations:** ^1^ Department of Family Medicine and Population Health, Virginia Commonwealth University, Richmond, Virginia, USA.; ^2^ Office of Research and Strategic Partnerships, Rhode Island School of Design, Providence, Rhode Island, USA.; ^3^ Institute for Women's Health, Virginia Commonwealth University, Richmond, Virginia, USA.; ^4^ Department of Obstetrics and Gynecology, and Virginia Commonwealth University, Richmond, Virginia, USA.; ^5^ Department of Psychiatry, Virginia Commonwealth University, Richmond, Virginia, USA.

**Keywords:** mental health, music, song writing, singing

## Abstract

***Background:*** This pilot study investigated the impact of a musical intervention on maternal/fetal attachment, psychiatric symptoms, and perceived stress in two centers.

***Materials and Methods:*** Forty-four pregnant women participated from the Virginia Commonwealth University in Richmond, VA, and Jacobi Medical Center in Bronx, NY. Participants were assigned to a lullaby intervention or control group. The Maternal Fetal Attachment Scale, Perceived Stress Scale (PSS), and Symptom Checklist (SCL-27) were completed at baseline and follow-up.

***Results:*** Although no significant differences were found in maternal/fetal attachment between control and intervention groups, there were within-group differences in both groups from baseline to follow-up. No statistically significant differences in change from baseline occurred on the SCL-27 and PSS.

***Conclusions:*** Exposure to a lullaby intervention was not statistically associated with maternal/fetal attachment, mental health, and perceived stress in this pilot study. Future studies with larger samples and different outcomes are suggested.

## Introduction

During pregnancy, symptoms of stress, anxiety, and depressed mood are common^1,2^ and often associated with risk factors, including low socioeconomic status, financial concerns, lack of social support, and younger and older maternal age.^3–5^ Stress in pregnancy bears clinical and public health relevance due to the association between high maternal stress and poor maternal/infant outcomes,^6^ including higher rates of preterm birth and analgesia use in labor.^1^ Maternal stress is also associated with poorer maternal/infant attachment^2^ and a subsequent diagnosis of antenatal and/or postnatal depression.^3,7^

Interventions focused on reducing maternal stress, anxiety, and depression in pregnancy and thus improving maternal/fetal outcomes are warranted. In particular, nonpharmacological-based interventions are desirable due to concerns about the impact of medication use on the developing fetus. The use of music and maternal singing during pregnancy represents one such nonpharmacological intervention that may be of benefit. Previous research has demonstrated preliminary evidence for some beneficial effects of exposure to music during pregnancy for both mother and the developing baby,^1,8–10^ including pain management during labor and birth, reduced stress, anxiety and depression,^9^ improved maternal/infant attachment,^11,12^ and improved adjustment among teenage mothers.

Over the last 7 years, Carnegie Hall's Weill Music Institute Lullaby Project pairs pregnant women and new mothers with professional musicians to write and sing personal lullabies for their babies, supporting maternal health, aiding child development, and strengthening the bond between parent and child. Since 2011, ∼1000 lullabies have been created globally in hospitals, correctional facilities, shelters, high schools, programs for teen parents, and other settings. The purpose of this music-based intervention is to encourage and support the bonding process, to support parents' aspirations to create the family they want for their children, and to harness their creativity as a tool for imagining and building future opportunities for healthy beginnings for their family. Preliminary insights from a 2-year qualitative analysis of the Lullaby Project by WolfBrown Associates^13^ suggest that lullabies may have the capacity to strengthen bonds between parents and children, and also provide an opportunity for parents to experience positive emotions, including feelings of competency, that may help promote resilience during difficult circumstances.

The transition to parenthood is a unique developmental phase that constitutes a period of stressful and sometimes maladaptive change for a significant proportion of new parents. Caring for an infant or young child can be taxing among the healthiest of parents, particularly in times of high stress. Adults who have experienced overwhelming and frightening events in their childhood, such as abuse and neglect, and/or adverse experiences in adulthood such as poverty or homelessness, are at higher risk for experiencing parenting challenges on a day-to-day basis. These adults are also at higher risk for reporting greater levels of parenting stress, which has been associated with parenting difficulties and poor developmental outcomes in children. Historically, intervention efforts aimed at improving developmental outcomes in children and families have been primarily focused on high-risk mothers and children, such as women with depression, substance abuse, trauma, and children exposed to severe sexual, physical abuse, or domestic violence. Intervention efforts have been less focused on treating vulnerable children and mothers identified as at-risk, but not high-risk, such as women with poor social support or economic hardship.

The purpose of the present pilot study was to investigate the potential impact of exposure to a brief musical intervention on maternal/fetal attachment, self-reported psychiatric symptoms, and perceived stress in a two-center, randomized-controlled trial of pregnant women at-risk for poor social support and economic hardship. Specifically, the study seeks to answer the following questions:

1. Does participation in the Lullaby Project intervention impact maternal/fetal attachment?
2. Are there differences in mental health outcomes based on participation in the Lullaby Project intervention?
3. Does participation in the Lullaby Project intervention reduce perceived maternal stress?

## Materials and Methods

### Participants

Research participants consisted of 44 pregnant women recruited from the Department of Obstetrics and Gynecology at Virginia Commonwealth University Health System in Richmond, VA, and Jacobi Medical Center in Bronx, NY. The sample size was determined based on the following: (1) feasibility with the timeline of 1 year to complete this pilot study, (2) appropriate sizing for the intervention group (*e.g.*, 8–12 women) based on prior preliminary results of lullaby sessions, and (3) ensuring balance between treatment and control groups. Eligibility criteria included being at least 18 years of age, pregnant in the second or third trimester, English speaking, and ability to provide informed consent. Exclusion criteria included being younger than 18 years and/or presenting with language barriers that limited one's ability to provide informed consent. This study was approved by the Institutional Review Board at both sites.

### Measures

The Maternal Fetal Attachment Scale (MFAS)^14^ is a 24-item Likert scale designed to measure the construct of maternal/fetal attachment during pregnancy. The instrument has five subscales that propose to measure aspects of the relationship between mother and fetus, which include the extent to which women engage in behaviors that represent affiliation and interaction with their unborn baby.

The Symptom Checklist (SCL-27-plus)^15^ is a short, multidimensional screening instrument for mental health problems. It contains five scales on current symptoms: depressive, vegetative, agoraphobic, and sociophobic symptoms and pain; a global severity index (GSI-27); a lifetime assessment for depressive symptoms; and a screening question for suicidality.

The Perceived Stress Scale (PSS)^16^ is the most widely used psychological instrument for measuring the perception of stress. It is the measure to the degree of situations in one's life that are appraised as stressful. Items include 10 questions that were designed to tap how unpredictable, uncontrollable, and overloaded respondents find their lives. The scale also includes a number of direct queries about current levels of experienced stress.

Demographics assessed participant age, sex, racial background, educational level, relationship status, number of prior pregnancies, number of living children, household income, and employment status.

### Procedure

Eligible participants were identified by their prenatal care clinician. A research team member described the study and provided interested potential participants with an IRB-approved flyer. The flyer provided a description of the study and a link to a secure online survey platform to complete screening questions to determine eligibility. Eligible participants were then asked to provide their name and phone number so that study staff could contact them to review the consent form, answer any questions they may have, and inquire about their consent to participate in this study.

#### Sampling and randomization

Women who consented to participate were randomly assigned in a 1:1 ratio to participate in an intervention or control group. The comparison group was necessary to account for possible biases arising from intervention effect and other related factors that may artificially impact the outcome variables being assessed. A randomization error occurred in our research database resulting in substantial imbalance between the groups such that all (91%) primiparous women were assigned to the intervention, while the control group constituted 100% multiparous women. All participants, regardless of which group they were assigned to, were provided with referral information for mental health support.

#### Baseline assessments at enrollment

Upon completion of consent during the second or third trimester, each participant was asked to complete a self-report battery of the MFAS, SCL-27, and PSS *via* the online secure platform. Participants could opt not to answer any questions they chose, and could elect to end the survey at any time.

#### Intervention

Women assigned to the intervention participated in one of two intervention groups, one at Virginia Commonwealth University (VCU) and one at Jacobi Medical Center. Each group participated in three group sessions, as described below, with ∼11–12 other pregnant women each group. The three group sessions were scheduled within a 3-week window in April–May of 2017.

1. Session 1: (5 hours) Participants collaborated with 1–2 project musicians to compose an original lullaby.
2. Session 2: (5 hours) Participants collaborated with project musicians to refine and record their lullaby.
3. Session 3: (2 hours) In a group format, all participants listened to each recorded lullaby and were invited to reflect on their experience. Examples of questions asked include, “What do you think of your song,” “did it change throughout the process,” and “how do you think you will use the song with your child and/or family?”

#### Postintervention assessments

To determine the potential impact of the intervention, women participating in the intervention group completed the same online questionnaires administered at baseline (with the exception of demographics) at the conclusion of Session 3 of the intervention group in May of 2017.

#### Control group

This study assessed outcomes for women who met the study eligibility criteria, but were not assigned to the intervention group during the second or third trimester. Participation for the control group took ∼1 hour and consisted of completing the online questionnaires on two occasions: upon enrollment and one follow-up online session around the same time as the conclusion of Session 3 of the intervention group in May of 2017.

The intervention and follow-up outcome measures were completed before childbirth. Participants who completed the study were compensated for their time.

### Data analysis

Data are presented as combined analyses for both sites. Each scale and its corresponding subscale were summarized using medians and ranges. The Wilcoxon signed-rank test was used to assess within-group differences and whether the median scores calculated for each instrument and subscale after Session 1 (baseline) were significantly different from the median scores calculated after Session 3 (postintervention) for women in the intervention group, and also for women in the control group. In additionally, for each scale and subscale, the Wilcoxon rank-sum test was used to evaluate whether the median scores for the intervention group were significantly different to those in the control group. All statistical analyses were completed in SAS v9.4 using a significance level of α = 0.05.

## Results

A total of 44 women were available for analysis; 23 women were assigned to the intervention group and 21 to the control group. The proportion of women who were white or Hispanic was equal to the proportion of women who identified as black (41.9%). Most women were unmarried (56.8%), had completed college (42.9%), and were unemployed (54.6%) (Table 1).

**Table 1.** Distribution of Study Sample Characteristics

Characteristics	Total	Intervention	Control	p^a^
	N = 44	N = 23	N = 21	
	n (%)	n (%)	n (%)	
Race				0.5093
White (includes Hispanics)	18 (41.9)	8 (34.8)	10 (50.0)	
Black	18 (41.9)	10 (43.5)	8 (40.0)	
Other	7 (16.3)	5 (21.7)	2 (10.0)	
Marital status				0.5702^b^
Married	19 (43.2)	9 (39.1)	10 (47.6)	
Other	25 (56.8)	14 (60.9)	11 (52.4)	
Education				0.5278
High school or less	11 (26.2)	5 (21.7)	6 (31.6)	
Some college	13 (31.0)	9 (39.1)	4 (21.1)	
College graduate or more	18 (42.9)	9 (39.1)	9 (47.4)	
Employment status				0.1466
Full-time	13 (29.6)	9 (39.1)	4 (19.1)	
Part-time	6 (13.6)	4 (17.4)	2 (9.5)	
Not presently employed	24 (54.6)	9 (39.1)	15 (71.4)	
Student	1 (2.3)	1 (4.4)	0 (0.0)	
Estimate of yearly income				0.7845
Less than $10,000	11 (25.0)	6 (26.1)	5 (23.8)	
$10,000 to less than $20,000	8 (18.2)	5 (21.7)	3 (14.3)	
$20,000 to less than $40,000	8 (18.2)	4 (17.4)	4 (19.1)	
$40,000 to less than $60,000	2 (4.6)	2 (8.7)	0 (0.0)	
$60,000 to less than $80,000	1 (2.3)	1 (4.4)	0 (0.0)	
$80,000 to less than $100,000	2 (4.6)	1 (4.4)	1 (4.8)	
$100,000 to less than $200,000	3 (6.8)	1 (4.4)	2 (9.5)	
Don't know/prefer not to answer	9 (20.5)	3 (13.0)	6 (28.6)	
First pregnancy				<0.0001^c^
Yes	21 (47.7)	21 (91.3)	0 (0.0)	
No	23 (52.3)	2 (8.7)	21 (100.0)	
No. of pregnancies				0.2220
Singleton	42 (95.5)	23 (100.0)	19 (90.5)	
Twins	2 (4.6)	0 (0.0)	2 (9.52)	
Reproductive technology use				0.2341
Yes	3 (6.8)	3 (13.0)	0 (0.0)	
No	41 (93.2)	20 (87.0)	21 (100.0)	
	**Mean (SD)**	**Mean (SD)**	**Mean (SD)**	***p***^**d**^
Age (years)	28.1 (4.6)	26.6 (4.6)	29.8 (4.1)	0.0206^c^
Gestational age (weeks)	24.5 (5.7)	24.0 (5.6)	25.2 (5.9)	0.4855

^a^ Fisher's exact test.

^b^ Chi-square test.

^c^ Indicates significance at α = 0.05 level.

^d^ Two-sample *t*-test.

SD, standard deviation.

Participants answered 100% of survey questions. A randomization error occurred in our research database such that all (91%) primiparous women were assigned to the intervention, while the control group constituted 100% multiparous women. The results for the intervention group showed that there was a significant difference between baseline and postintervention median MFAS score (*p*-value = 0.0166). For the control group, there was a statistically significant difference in the MFAS baseline and follow-up medians (*p*-value = 0.0396), and the “dysthymic symptoms” and “symptoms of mistrust” subscales of the SCL-27-plus scales (*p*-value = 0.0430 and *p*-value = 0.0313, respectively). There were no statistically significant differences in median change from baseline between the intervention and control group with the exception of the “symptoms of mistrust” subscale of the SCL-27-plus scales (*p*-value = 0.0315) (Table 2).

**Table 2.** Mean Change from Baseline Between the Intervention and Control Group

	Intervention group			Control group			p^b^
	Baseline	Postintervention		Baseline	Postintervention	p^a^	
	Median [range]	Median [range]	p^a^	Median [range]	Median [range]		
Maternal/fetal attachment score	99.0 [81.0–114.0]	107.0 [91.0–112.0]	0.0166^c^	91.0 [77.0–104.0]	99.0 [82.0–113.0]	0.0396^c^	>0.99
Subscales							
Differentiation of self	17.0 [12.0–20.0]	17.0 [13.0–20.0]	0.9375	16.0 [12.0–20.0]	15.0 [13.0–18.0]	0.8066	0.9527
Interaction with the fetus	18.0 [13.0–23.0]	19.0 [13.0–22.0]	0.4600	16.0 [10.0–21.0]	18.0 [9.0–24.0]	0.1074	0.5101
Attributing characteristics to the fetus	23.0 [18.0–30.0]	26.0 [22.0–30.0]	0.0215^c^	22.0 [14.0–27.0]	22.0 [17.0–30.0]	0.1582	0.5177
Giving of self	22.0 [15.0–25.0]	22.0 [20.0–25.0]	0.2813	20.0 [14.0–24.0]	23.0 [17.0–25.0]	0.1748	0.8491
Role taking	20.0 [14.0–20.0]	20.0 [15.0–20.0]	0.7500	18.0 [15.0–20.0]	20.0 [14.0–20.0]	0.1367	0.0919
PSS	20.0 [9.0–34.0]	18.0 [10.0–29.0]	>0.99	23.0 [7.0–35.0]	15.0 [7.0–28.0]	0.4053	0.5430
SCL-27	38.0 [27.0–84.0]	45.0 [28.0–68.0]	0.4465	37.0 [27.0–87.0]	32.0 [27.0–66.0]	0.0762	0.1131
Subscales							
Depressive symptoms	5.0 [4.0–14.0]	6.0 [4.0–9.0]	0.3867	5.0 [4.0–8.0]	5.0 [4.0–10.0]	0.8633	0.6413
Dysthymic symptoms	7.0 [4.0–17.0]	8.0 [4.0–17.0]	0.7446	8.0 [4.0–19.0]	5.0 [4.0–16.0]	0.0430^c^	0.1414
Vegetative symptoms	10.0 [6.0–18.0]	8.0 [6.0–23.0]	0.7227	8.0 [6.0–21.0]	8.0 [6.0–18.0]	0.9141	0.8772
Agoraphobic symptoms	6.0 [5.0–13.0]	6.0 [5.0–13.0]	0.3125	6.0 [5.0–16.0]	5.0 [5.0–9.0]	0.1777	0.0772
Social phobia symptoms	5.0 [4.0–12.0]	7.0 [4.0–8.0]	0.6328	6.0 [4.0–12.0]	5.0 [4.0–8.0]	0.1250	0.2598
Symptoms of mistrust	4.0[4.0–16.0]	6.0 [4.0–11.0]	0.5547	5.0 [4.0–13.0]	4.0 [4.0–8.0]	0.0313^c^	0.0315^c^

^a^ Wilcoxon signed-rank test (within-group differences).

^b^ Wilcoxon rank sum test (between-group differences).

^c^ Indicates significance at α = 0.05 level.

PSS, Perceived Stress Scale; SCL-27, Symptom Checklist.

## Discussion/Conclusions

Music has the power to build a sense of community and belonging. The purpose of this music-based intervention is to encourage and support the bonding process, to support parents' aspirations to create the family they want for their children, and to harness their creativity as a tool for imagining and building future opportunities for healthy beginnings for their family. This pilot study builds on the foundational qualitative data obtained previously and represents the first multisite study examining the potential impact of this brief musical intervention on measured outcome variables. Together, these features allow the current study to address methodological limitations obtained from a purely qualitative approach. Overall, our findings indicated that exposure to a brief lullaby intervention was not associated with statistical differences with regard to maternal/fetal attachment, maternal mental health, and perceived stress in this pilot study.

Although there is interest in the relationship between music and social bonding, there is no current consensus about the mechanisms by which this might occur. It has been argued that group-music making leads to social bonding due to the release of neurohormones, specifically oxytocin,^17–19^ although such explanations lack robust evidence. Other studies have investigated activation of the endogenous opioid system through music. Additional research related to potential mechanistic theories is needed.

Regarding maternal/fetal attachment, no statistically significant differences emerged between the intervention and control groups. However, both groups showed a significant difference between baseline and postintervention median MFAS scores, with intervention participants demonstrating a more marked increased, as expected. These findings are consistent with the literature about mother/newborn bonding, suggesting a progressive increase in the level of bonding from birth onward. Indeed, a recent quasirandomized study examining maternal singing during pregnancy and 3 months after birth found no significant differences with regard to prenatal attachment; in contrast, postnatal bonding was significantly greater in the singing group 3 months after birth.^20^ These results add to existing evidence demonstrating a beneficial impact of maternal singing after birth. Future research efforts may benefit from using longitudinal methodology across pregnancy and during the postpartum period.

We recognize that our study has limitations. One study challenge that occurred was a randomization error in our research database such that all (91%) primiparous women were assigned to the intervention, while the control group constituted 100% multiparous women. This represents a statistical error and serious methodological limitation that led to an imbalance in the baseline characteristics of the two groups. It may also have biased the data itself.

Mothers in the lullaby intervention reported experiencing positive emotions while singing. This has been supported elsewhere, including maternal report that the act of singing enriches the relationship new mothers have with their babies.^20^ It is possible that subjective ratings of maternal emotions while singing lullabies and the feelings mothers self-report in relation to their babies when they engage in singing may, in fact, differ from more objective and face-valid assessment measures of these constructs. The process of inviting mothers to reflect on their thoughts and feelings composing, recording, and listening to their original lullaby can inform future qualitative research. More attention to these issues is needed.

The small sample size could have contributed to the lack of statistical differences with regard to the outcome variables in this study. Moreover, it is possible that the instruments selected may not have measured the true impact and different outcome variables might have produced findings of statistical difference. It may also have been helpful to assess postpartum outcomes and to measure generalizability if intervention participants actually used the lullaby at home with their baby. This pilot study replicated the lullaby project in two public hospitals. Future studies with larger samples, validated measures, mothers at different stages of parenting, expanding inclusion to family members, and different outcomes are suggested.

### Implications for research and policy and/or practice

Historically, intervention efforts aimed at improving developmental outcomes in children and families have been primarily focused on high-risk mothers and children, such as women with depression, substance abuse, trauma, and children with severe behavioral or emotional difficulties. Intervention efforts have been less focused on treating vulnerable families identified as at-risk, but not high-risk, such as women with poor social support or economic hardship. This is an urgent public mental health issue because these at-risk families should receive support before they become high-risk or are identified as having developmental risk. Although there appears to be general consensus regarding the importance of intervening with young children and their parents in early childhood in particular, these families often elude early detection and intervention. There is compelling evidence for working within prevention and early intervention models of health for this population. Increased scientific attention is warranted to conduct prevention-related research aimed at better understanding developmental processes to influence developmental outcomes for young children and their parents, and to establish needs for ongoing research and its application to policy.

## Abbreviations Used

MFAS: Maternal Fetal Attachment Scale
PSS: Perceived Stress Scale
SCL-27: Symptom Checklist
SD: standard deviation
VCU: Virginia Commonwealth University

## References

[1] Alder J, Fink N, Bitzer J, Hösli I, Holzgreve W Depression and anxiety during pregnancy: A risk factor for obstetric, fetal and neonatal outcome? A critical review of the literature. J Matern Fetal Neonatal Med 2007;20189–209 10.1080/14767050701209560 17437220

[2] Monk C, Leight KL, Fang Y The relationship between women's attachment style and perinatal mood disturbance: Implications for screening and treatment. Arch Womens Ment Health 2008;11:117–129 18493708 10.1007/s00737-008-0005-x PMC4472000

[3] Britton JR Maternal anxiety: Course and antecedents during the early postpartum period. Depress Anxiety 2008;25:793–800 17397041 10.1002/da.20325

[4] Cunningham M, Zayas LH Reducing depression in pregnancy: Designing multimodal interventions. Soc Work 2002;47:114–123 12019798 10.1093/sw/47.2.114

[5] Lancaster CA, Gold KJ, Flynn HA, Yoo H, Marcus SM, Davis MM Risk factors for depressive symptoms during pregnancy: A systematic review. Am J Obstet Gynecol 2010;202:5–14 20096252 10.1016/j.ajog.2009.09.007 PMC2919747

[6] Cook N, Ayers S, Horsch A Maternal posttraumatic stress disorder during the perinatal period and child outcomes: A systematic review. J Affect Disord 2018;225:18–31 28777972 10.1016/j.jad.2017.07.045

[7] Heron J, O'Connor TG, Evans J, Golding J, Glover V AL SPAC Study Team The course of anxiety and depression through pregnancy and the postpartum in a community sample. J Affect Disord 2004;80:65–73 15094259 10.1016/j.jad.2003.08.004

[8] Arya R, Chansoria M, Konanki R, Tiwari DK Maternal music exposure during pregnancy influences neonatal behaviour: An open-label randomized controlled trial. Int J Pediatr 2012;2012:901812 22518187 10.1155/2012/901812 PMC3299264

[9] Chang M-Y, Chen C-H, Huang K-F Effects of music therapy on psychological health of women during pregnancy. J Clin Nurs 2008;17:2580–2587 18298503 10.1111/j.1365-2702.2007.02064.x

[10] Partanen E, Kujala T, Tervaniemi M, Huotilainen M Prenatal music exposure induces long-term neural effects. PLoS One 2013;8:e78946 24205353 10.1371/journal.pone.0078946 PMC3813619

[11] Blumenfeld H, Eisenfeld L Does a mother singing to her premature baby affect feeding in the neonatal intensive care unit? Clin Pediatr (Phila) 2006;45:65–70 16429218 10.1177/000992280604500110

[12] Cevasco AM The effects of mothers' singing on full-term and preterm infants and maternal emotional responses. J Music Ther 2008;45:273–306 18959452 10.1093/jmt/45.3.273

[13] Wolf DP Lullaby: Being Together, Being Well. New York: Carnegie Hall's Weill Music Institute, 2017:1–24.

[14] Cranley MS Development of a tool for the measurement of maternal attachment during pregnancy. Nurs Res 1981;30:281–284 6912989

[15] Hardt J, Gerbershagen HU Cross-validation of the SCL-27: A short psychometric screening instrument for chronic pain patients. Eur J Pain 2001;5:187–197 11465984 10.1053/eujp.2001.0231

[16] Yokokura AVCP, Silva AAM da, Fernandes J de KB, et al. Perceived Stress Scale: Confirmatory factor analysis of the PSS14 and PSS10 versions in two samples of pregnant women from the BRISA cohort. Cad Saude Publica 2017;33:e00184615 29267695 10.1590/0102-311X00184615

[17] Freeman WJ III A neurobiological role of music in social bonding, in the origins of music. UC Berkeley: MIT Press, 1998

[18] Grape C, Sandgren M, Hansson L-O, Ericson M, Theorell T Does singing promote well-being?: An empirical study of professional and amateur singers during a singing lesson. Integr Physiol Behav Sci 2003;38:65–74 12814197 10.1007/BF02734261

[19] Huron D Is music an evolutionary adaptation? Ann N Y Acad Sci 2001;930:43–61 11458859 10.1111/j.1749-6632.2001.tb05724.x

[20] Persico G, Antolini L, Vergani P, Costantini W, Nardi MT, Bellotti L Maternal singing of lullabies during pregnancy and after birth: Effects on mother-infant bonding and on newborns' behaviour. Concurrent Cohort Study. Women Birth 2017;30:e214–e220 28169158 10.1016/j.wombi.2017.01.007

